# Review of Infections With Bovine Herpesvirus 1 in Slovenia

**DOI:** 10.3389/fvets.2021.676549

**Published:** 2021-07-01

**Authors:** Peter Hostnik, Danijela Černe, Janko Mrkun, Jože Starič, Ivan Toplak

**Affiliations:** ^1^Institute for Microbiology and Parasitology-Virology Unit, Veterinary Faculty, University of Ljubljana, Ljubljana, Slovenia; ^2^Clinic for Reproduction and Large Animals-Clinic for Reproduction, Veterinary Faculty, University of Ljubljana, Ljubljana, Slovenia; ^3^Clinic for Reproduction and Large Animals-Section for Ruminants, Veterinary Faculty, University of Ljubljana, Ljubljana, Slovenia

**Keywords:** BoHV-1, Slovenia, antibody detection, insemination centres, monitoring

## Abstract

In the 1950s, infectious bovine rhinotracheitis/infectious pustular vulvovaginitis (IBR/IPV) disease was clinically detected and documented in cattle for the first time in Slovenia. The bovine herpes virus 1 (BoHV-1) was confirmed several times from infected herds by virus isolation on cell cultures. To keep the IC virus-free, high biosecurity measures were introduced. Before entering the IC, all calves are serologically tested and quarantined. Bulls in Slovenian insemination centres (IC) have been negative for IBR /IPV infection since 1979. From 1985 to 1991, few large-scale studies of the prevalence of IBR/IPV were carried out. In 1985, a high percentage (56.9%) of serologically positive animals were found in large state farms with Holstein Friesian cattle. Epidemiological studies in farm with bulls' mother herds were also carried out in the farms with Simmental and Brown cows. Antibodies against BoHV-1 were detected in the serum of 2.3% of Brown cattle and 3.5% of Simmental cattle. In the year 2000, 3.4% of bulk tank milk samples from 13,349 dairy farms were detected BoHV-1 antibodies positive. The highest percentage of positive animals was found in regions with an intensive grazing system (6.2% positive) and the lowest percentage in the east part of Slovenia (0.9% positive) on farms with mostly Simmental cattle. In 2006, a total 204,662 sera of cattle older than 24 months were tested for the presence of BoHV-1 antibodies and positive cattle were detected in 3.6% of tested farms. These farms kept 34,537 animals that were potential carriers of the BoHV-1. Most of the positive farms kept Holstein Friesian cattle, descendants from the state-owned farms, which were privatised or closed after 1990. In 2015, the Administration of the Republic of Slovenia for Food Safety, Veterinary and Plant Protection issued a rule that describes the conditions for granting and maintaining the status of BoHV-1 free holdings. The rule provides a voluntary control programme for breeders who want to obtain BoHV-1 free status and are willing to cover all the cost of acquiring and maintaining that status. There has been very little response from breeders.

## Introduction

Bovine alphaherpesvirus 1 is a member of the genus *Varicellovirus*, subfamily *Alphaherpesvirinae* in the family *Herpesviridae* (https://talk.ictvonline.org/taxonomy/), known as Bovine herpesvirus 1 (BoHV-1) or infectious bovine rhinotracheitis (IBR) infectious pustular vulvovaginitis (IPV) virus. It cause severe economic losses in livestock (1, 2). It is classified into three subtypes (BoHV-1.1, BoHV-1.2a, and BoHV-1.2b), which are associated with respiratory disease (rhinotracheitis, pneumonia) and other severe conditions such as, vulvovaginitis, balanoposthitis, conjunctivitis, genital lesions, reproductive disorders, abortions, encephalitis, and general infections (3–5). Clinical signs vary from severe and fatal to mild and even subclinical, and outcomes are dependent on combinations of viral, host, and environmental factors. Infections also cause transient immunosuppression, which, together with damage to the respiratory mucosa, makes BoHV-1 an important pathogen in Bovine Respiratory Disease Complex (BRDC), the most important respiratory disease in cattle (1, 6).

After infection, replication of the virus at the primary site of entry, usually the respiratory or genital mucosa, followed by infection of sensory nerve endings. BoHV-1 is then transported by retrograde axonal flow to the ganglia or tonsils, where it remains in a latent form (7). BoHV-1 is difficult to detect when is in latent form (7). Infection with BoHV-1 virus usually results in the lifelong- presence of specific antibodies. However, some infected animals contain very low quantity of BoHV-1 antibodies. Even a seronegative animal can be a latent carrier of the virus in the case when maternal antibodies can interfere with a humoral immune responses following infection or vaccination (8, 9). Latently infected animals shed less virus, they can still infect others and therefore it can be detected. Reactivation and shedding of virus is a distinct factor in the epidemiology of BoHV-1 (10).

BoHV-1 is commonly shed with bovine semen. Viral load found in bovine semen from naturally infected bulls ranged from 10^1.5^ to 10 ^5.0^ TCID_50_/50 μl. The virus is also known to be the most frequently present in the seminal fluid fraction (10). Virus may be shed through mucous membrane of either upper respiratory, genital tract or conjunctival epithelium. Usual routes of transmission of BoHV-1 are nose to nose contact, with droplets on short distances and by mating. Genital transmission of BoHV-1 also occurs through infected bull semen by artificial insemination (AI). In this manner, the virus can be transmitted to large numbers of cows and may cause miscarriages, infertility, endometritis, and embryonic death. Annual systematic individual screening of bull in insemination centres (IC) for BoHV-1 antibodies and rearing under quarantine conditions may ensure the use of BoHV-1-free semen. In Slovenia, bovine semen is collected only in ICs which are free from IBR/IPV (11).

In the European Union (EU), several countries or regions are considered BoHV-1 free, following the implementation of EU-approved eradication programmes, including Austria, Germany, Denmark, Finland, Sweden, Jersey (United Kingdom), Valle d'Aosta, the Province of Bolzano (Italy), and the Czech Republic, as of 2020 (12). Belgium, Luxembourg, France, and the region of Friuli-Venezia Julia in Italy have approved an eradication programme according to Article 9 of Directive 64/432/EEC [(13); new Animal Health Law it's applicable from 21. April 2021]. The Netherlands has a compulsory eradication programme for dairy herds and a voluntary programme for non-dairy herds (14), which is not in accordance to Directive 64/432/EEC.

In Slovenia, which has a total area of 20,271 km^2^, at the end of 2019, 466,911 cattle were registered in 29,615 holdings: 98.3% on family farms and 1.7% on agricultural enterprises (formerly state-owned). An average Slovenian holding reared 15.8 animals. In 2018 the 29.9% of the animals were of the Simmental breed, 16.8% Holstein, 4.4% Brown, and 0.9% of autochthonous Cika breed. The rest of the animals (48.0%) were either crossbred, animals with unknown pedigree, or beef breeds (mostly Simmental, Limousin, Charolais, or Angus). Among animals, cows predominate (34.0%), followed by calves (29.8%), heifers (20.8%), and bulls (15.4%) (15). In 1985, 57,7206 cattle, mainly Simmental (55.1%), Brown (31.2%), Holstein Friesian (8.8%), and others (3.9%) were bred. State farms reared 9,412 (5.5%) cows, mostly Holstein Friesian breed, other dairy cows were bred by family farms (16).

BoHV-1 has been clinically detected in Slovenia in 1950s. The virus was isolated for the first time on bovine kidney primary cell culture and antibodies detected by a virus neutralisation test in 1967 (17, 18).

The first phase of control of BoHV-1 infection was based on the monitoring of BoHV-1 antibodies in ICs and breeding centres for young bulls (BCYB). All bulls in both ICs were serologically tested twice a year for BoHV-1 antibodies. Soon there was a problem with obtaining Holstein Friesian calves for BCYB with a serologically negative result. Since 1979, only serologically negative bulls from serologically negative cows have been admitted to BCYBs, except for Holstein Friesian bulls, since it was not possible to obtain enough breeding bulls from few BoHV-1 negative herds. The top Holstein Friesian bull's mothers in state farms which were BoHV-1 positive, all bull's mothers were vaccinated against BoHV-1 by conventional vaccines according to the manufacturer's instructions to prevent the spread of infection (11, 19). Only in the event that the cow and her calf reacted serologically negatively was the calf allowed to enter the quarantine barn in BCYB.

Imported semen was also under laboratory control. All BoHV-1 antibody positive bulls, as well as young bulls in BCYBs, were culled. The last seropositive bull in IC was found in 1979. In the same year, BoHV-1 was isolated from bull semen imported from the USA (20).

In 1990, when there were already enough BoHV-1 negative herds with bull's mothers, the programme for selecting calves for BCYB was changed. With regard to the protection of ICs and bulls, an agreement was reached in 1995 together with the Veterinary administration and Livestock Selection service of Slovenia that all bull's mother herds should be serologically tested every year and only calves from BoHV-1-negative bull's mother herds may be admitted to BCYB (21). State farms with Holstein Friesian cattle with seropositive animals were excluded from bull's mother herds (21).

Until 2003, bull's mother herds were subject to annual monitoring for the presence of antibodies to BoHV-1. The testing included holdings designated by the Livestock Selection Service of Slovenia. Blood samples were taken from all categories of animals over 6 months old.

Since 2004, the breeding of bull mothers is no longer under the annual inspection of all animals in the herd for BoHV-1 antibodies. The process of housing calves in BCYBs changed slightly. After approval of a genetically suitable calf for a breeding bull candidate, a blood sample is taken from the calf and its mother before the calf is transferred to the BCYB at the age of 4 months or less and tested for the presence of BoHV-1 antibodies. If both calf and his mother are negative, the calf is moved to the quarantine of BCYB. During quarantine, the calf is re-tested to the presence of BoHV-1 antibodies. If the result is negative for antibodies against BoHV-1, the calf may enter the BCYB. At the age of 15 months, a quality assessment of bulls carried out; for example, the andrological examination and examination for the presence of BoHV-1 antibodies. Only the best bulls go to the ICs and the rest to natural mating. A bull always enters the IC after 30 days of quarantine and re-examination for the presence of BoHV-1 antibodies is done once per year.

The structure of farms in Slovenia changes from year to year. The number of cattle in the country is stable, but the number of herds is declining rapidly, and those that remain are increasing the number of cattle. Limited data are available on the impact of BoHV-1 in such a system, and it is important to examine whether the effect of BoHV-1 in Slovenia is similar to that reported previously in more intensive livestock systems.

This article presents a historical overview of the work in the field of control of BoHV-1 virus infections in Slovenia and the veterinary service's efforts to keep breeding bulls free of this infection and the adoption of a mandatory eradication programme. The aim of this paper is chronologically to present the results of all previous studies on prevalence of BoHV-1 in Slovenia, which should help decision-makers in preparing a program for the compulsory eradication of BoHV-1 in Slovenia.

## Materials and Methods

### BoHV-1 Infections From 1976 to 1984

Between 1976 and 1984, 6,101 blood samples from cattle were tested for antibodies to the BoHV-1. Cattle included into testing were bulls in ICs and young bulls in BCYBs, predominantly calves and bull's mothers, of which 3,403 were from state dairy farms, ICs and BCYBs and 2,698 cattle from private breeders. Sera were tested for the presence of antibodies to the BoHV-1 virus in a virus neutralisation test (VNT) in accordance with the O.I.E. standards. In microplate wells (Nunc), 2-fold dilutions of test sera (1:2 and 1:4) were prepared. Working dilutions of the reference BoHV-1 strains ZRG (100–500 TCID_50_/0.05 ml), were added to the serum dilutions in the same amount (0.05 ml). After 2 h of incubation of the virus-serum mixture at 37°C, 0.05 ml of a suspension of cells of the AUBEK cell line was added to all microplate wells. Serum and virus controls were also included in the test. The test was read on day 3 of microplate incubation at 37°C. A serum that neutralised the cytopathic effect of BoHV-1 in a 1:2 dilutions was declared positive.

### BoHV-1 Infections From 1985 to 2005

The monitoring for the identification of BoHV-1 negative bull's mother herds started in 1985 with Holstein Friesian breed of the state-owned herds, and in the following years continued with herds of other breeds, which were in private sector herds. In 1985, 4,291 cattle from ten different state herds were included. The investigation covered all categories of animals older than 6 months. In 1989, 3,837 cattle from 173 private herds of Simmental bull's mothers were tested for the presence of BoHV-1 antibodies. In 1990, 2,602 brown cattle bull's mothers were tested. Until 1991, the blood serum samples were tested by VNT and later Svanova ELISA kit (Svanovir^®^ IBR-Ab, Svanova, Uppsala, Sweeden) was used. The method is accredited according to the standard ISO17025.

After the independence of Slovenia in 1991, the number of imported cattle increased. In period from 1991 to 1995, 46,237 bovine sera were collected from quarantined imported cattle and were tested for BoHV-1 antibodies by Svanova ELISA kit. After 1995, when Slovenia joined the EU mandatory quarantines were banned for cattle imported from European countries.

The next epizootiological analysis for the purpose of protection of ICs free of BoHV-1 infection was performed in 1993. Eight thousand two hundred and eighty-one bovine animals older than 6 months selected by the Slovenian Selection Service for Cattle Breeding were examined in 327 private sector herds. The next attempt to find suitable BoHV-1 negative herds was made in 1995. In this campaign, 4,880 cattle were tested in 207 farms. From 1996 to 2003, all herds with bull's mothers were under annual serological control for the presence of BoHV-1 antibodies. Thus, from 6,205 cattle in 1996 to 14,704 cattle in 2003 were tested from bull's mothers herds and those which applied for this status. For the purpose of preparing the BoHV-1 eradication program for the entire country, the first major BoHV-1 monitoring was done in 2000. Bulk tank milk (BTM) samples were tested for the presence BoHV-1 antibodies in all 13,349 dairy farms (4.7 cows per herd on average) producing milk for public consumption.

### BoHV-1 Infections From 2006 to 2020

For the purpose of preparing the BoHV-1 eradication program for the entire country, the second major BoHV-1 monitoring was done in 2006. Blood samples, which were taken to obtain the status of a bovine brucellosis-free country, were tested also for BoHV-1 antibodies. The investigations covered 204,662 cattle from 35,991 farms, representing 79.9% of farms in Slovenia. Blood samples were taken from all cattle over 24 months of age. A total of 37,366 pools of up to 10 sera were prepared from individual serum samples according to the instructions of the manufacturer of the used kit Svanovir IBR ELISA ab test (Svanova, Uppsala, Sweden). In the case that animals from several herds were pooled and the pool was positive, the herds were retested individually. Since 2006, bull's mothers and their calves have been regularly monitored for the presence of BoHV-1 antibodies before being transferred to a BCYB, but not other cattle in bull's mother herds. Beside ICs and BCYBs bulls, the remaining samples that were analysed in this period were taken from animals that participated e.g., in the shows and individual blood samples, which were taken at the time of cattle selling. Only from 1,001 to 2,062 samples per year were tested for BoHV-1 antibodies in this period, of which 554–622 belonged to IC and BCYB bulls each year. All laboratory tests for detecting antibodies and BoHV-1 virus in Slovenia are performed in the Virology Laboratory of the National Veterinary Institute in Ljubljana. Since 1991, we have been using an accredited commercial ELISA test, Svanovir^®^ IBR-Ab (Svanova, Uppsala, Sweden).

### BoHV-1 Infections in IC and BCYB

All bulls in ICs and BCYBs have been tested to BoHV-1 antibodies yearly since 1976. Calves intended for BCYBs were serologically examined before entering and once again in the quarantine of the BCYBs. In addition, young bulls before entering ICs are quarantined and serologically examined.

## Results

### Surveillance of BoHV-1 Infections From 1976 to 1984

The percentage of positive cattle for BoHV-1 antibodies from 1976 to 1984 varied from 2.4 to 39.6% in state and from 2.2 to 6.1% in private herds. Yearly results are shown in Table 1.

**Table 1:** Results of the BoHV-1 serological analysis in Slovenia in the period 1976–1984.

**Year**	**State farms**			**Private herds**		
	**No. of samples**	**No. of positive**	**Positive (%)**	**No. of samples**	**No. of positive**	**Positive**
1976	197	22	11.6	ND	ND	ND
1977	252	6	2.4	ND	ND	ND
1978	171	8	4.6	ND	ND	ND
1979	642	154	33.3	586	18	3.0%
1980	349	18	5.1	152	9	5.9%
1981	354	38	11.8	453	10	2.2%
1982	407	96	23.6	341	21	6.1%
1983	389	31	7.9	580	7	1.3%
1984	642	254	39.6	586	18	3.1%
Total:	3,403	627	18.42	2,698	83	3.1%

### Surveillance of BoHV-1 Infections From 1985 to 2005

In 1985, within herd sero-prevalence ranging from 23.1 and 65.4% was found on six randomly selected BoHV-1-positive state farms with Holstein Friesians (Table 2). The infection was present in all state dairy farms. In 1989, only 3.5% of serologically positive Simmental cattle in 15 herds with bull's mothers were found. In 1990, 2.3% of Brown cattle in 11 farms were seropositive.

**Table 2:** BoHV-1 seroprevalence in cattle herds with bull's mothers among three most prevalent breeds in Slovenia.

**Year**	**Breed**	**No. of samples**	**Positive samples**	**Percentage of positive**	**No. of farms**	**Positive farms**	**Percentage of positive**
1985	Holstein Friesian	4,291	2,445	56.9%	6	6	100%
1989	Simmental	3,837	133	3.5%	137	15	10.9%
1990	Brown	2,602	61	2.3%	121	11	9.1%

Since independence of Slovenia in 1991, imported cattle had been quarantined and tested for BoHV-1 antibodies; results are shown in Table 3. In 1993, a study of the prevalence of BoHV-1 in herds with bull mothers was done. Out of 8,281 cattle, 281 seropositive cattle were found in 38 herds (Table 3). Of the 327 herds tested for BoHV-1 infection, 289 (88.4%) herds with bull mothers were declared free based on the results of serological tests. A low percentage of serologically positive animals (below 5%) was found in 14 of 38 BoHV-1 positive farms. In three herds, however, over 80% of animals reacted positively to BoHV-1 antibodies. Only one BoHV-1-positive animal was found in 16 farms. The highest percentage of serologically positive animals in a herd was 57 out of 66 (86.4%).

**Table 3:** Results of serological tests for BoHV-1 antibodies in imported cattle and bulls' mother herds in Slovenia in the period 1991–1995.

	**Imported cattle**			**Bull's mothers herds**		
**Year**	**No. of samples**	**IBR/IPV positive**	**Percentage of positive**	**No. of samples**	**IBR/IPV positive**	**Percentage of positive**
1991	530	314	59.2%	2.353	60	2.5%
1992	7,165	5.056	42.7%	ND	ND	ND
1993	4,599	2.181	47.5%	8.281	281	3.4%
1994	17,450	6.644	32.4%	ND	ND	ND
1995	16,493	2.892	17.5%	4.880	1.251	25.6%
Total	46,237	17.087	36.9%	15.514	1.592	10.3%

In 1995, 4,880 blood samples from 207 herds with bull mothers were tested. BoHV-1-positive animals were confirmed in 11 herds, of which only one herd that was negative in 1993 was positive, and the other 10 BoHV-1-positive herds were tested for the first time. From 1996 to 2003, 181 to 311 herds with bull mothers and candidates for herds with bull mother were included in yearly serological surveillance (Table 4).

**Table 4:** Results of BoHV-1 serological surveillance in herds with bull's mothers in Slovenia from 1996 to 2003.

**Year**	**No. of bull's mother herds**	**Number of BoHV-1 positive herds**	**Percentage of positive herds**	**Number of sera**	**Number of BoHV-1 positive cattle**	**Percentage of positive cattle**
1996	276	5	1.8%	6,205	75	1.2%
1997	248	18	7.3%	6,580	59	0.9%
1998	181	4	2.2%	5,366	7	0.1%
1999	265	7	2.6%	8,722	54	0.6%
2000	275	6	2.2%	10,603	146	1.4%
2001	309	2	0.6%	12,885	4	0.1%
2002	301	5	1.7%	13,686	22	0.2%
2003	311	15	4.8%	14,704	165	1.1%
Total:	2,166	77	3.5%	78,751	532	0.7%

In a serological study in commercial dairy farms on BTM samples in the year 2000 (Table 5), the highest percentage of positive herds was found in Gorenjska (6.1% of herds) and the lowest in Prekmurje (0.9% of herds). BoHV-1 positive samples were recorded in 447 herds, representing 3.4% of all tested herds. In 109 BoHV-1 positive herds, the cattle were predominantly of the Holstein Friesian, in 74 Simmental, and in 33 Brown breed. In the other 231 farms, there were animals of different breeds, of which 206 farms kept at least one Holstein Friesian cattle.

**Table 5:** Results of the study on the presence of BoHV-1 antibodies in bulk milk samples (BTM) in dairy herds producing milk for public consumption in Slovenia in the year 2000.

**Region**	**Number of herds**	**Positive herds**	**Percentage of positive herds**
Gorenjska	1,286	78	6.1%
Ljubljanska	1,611	71	4.4%
Primorska	1,126	21	1.9%
Celjska	1,569	60	3.8%
Mariborska	1,845	70	3.8%
Prekmurje	3,400	31	0.9%
Ptujska	1,050	56	5.3%
Dolenjska	1,462	60	4.1%
Total:	13,349	447	3.4%

### Surveillance of BoHV-1 Infections From 2006 to 2020

**Figure 1:**
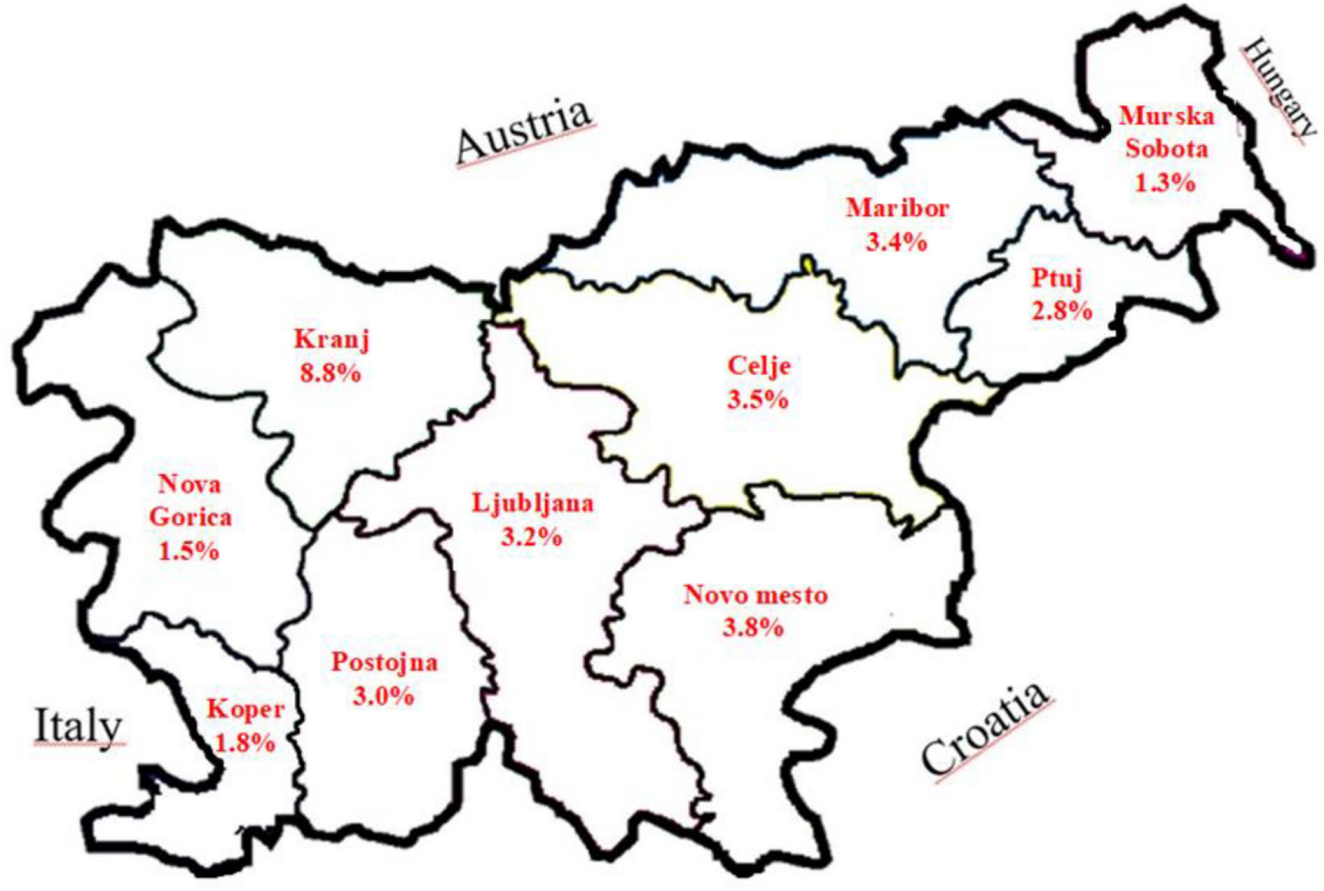
Prevalence of BoHV-1 positive herds by administrative regions in Slovenia in 2006.

In 2006, serum samples of 204,662 cattle older than 24 months were tested for the presence of antibodies against IBR/IPV; 79.9% of Slovenian herds were included in this investigation. BoHV-1-positive cattle were detected in 1,287 (3.6%) herds. The highest herd prevalence was recorded in the region Kranj (8.8%), and the lowest in the region Murska Sobota (1.3%) (Figure 1). Herds who also had Holstein Friesian cattle were positive most frequently among all herds. These herds were most often descendants of animals from previous state farms.

From 2007 to 2020, bulls from ICs and BCYBs were tested systematically and at the breeder's request also other animals (show animals and individual animals purchased and sold). Percentages of positive samples were from 0.1% in 2020 to 9.7% in 2015 (Table 6). All samples of bulls from ICs and BCYBs were negative for BoHV-1 antibodies from 2007 to 2020. Samples with positive results belonged to cattle intended for sale or exhibitions, etc.

**Table 6:** Number of samples for tests for BoHV-1 antibodies including samples of bulls from ICs and BCYBs in Slovenia in the period 2007–2020.

**Year**	**No. of samples**	**Number of BoHV-1 positive samples**	**Percentage of positive samples**
**Results of tests for the presence of antibodies against BoHV-1**			
2007	2,062	46	2.2%
2008	1,565	21	1.3%
2009	1,464	18	1.3%
2010	1,106	19	1.6%
2011	1,139	37	3.3%
2012	1,269	14	1.1%
2013	1,797	59	3.3%
2014	1,550	37	2.4%
2015	1,411	138	9.8%
2016	1,234	15	1.2%
2017	1,259	33	2.6%
2018	1,550	26	1.7%
2019	1,215	7	0.6%
2020	1,001	1	0.1%
Total	19,622	471	2.4%

### BoHV-1 Infections in ICs and BCYBs

Since 1979 all bulls in ICs have been negative to BoHV-1 antibodies with the exception in 1990. An outbreak of BoHV-1 was detected in the BCYB in Murska Sobota. During the regular serological monitoring of young bulls, 12 of 89 bulls from BCYB had BoHV-1 antibodies. There were 28 breeding bulls in a nearby IC, and 9 bulls had BoHV-1 antibodies. No clinical signs of infection were observed. All animals from the BCYB and IC were immediately culled. The source of virus introduction and infection was never determined.

## Discussion

The first IC with breeding bulls in Slovenia was established at the end of the 1950s, and the collection of bull semen and AI was introduced, primarily in response to sexually transmitted diseases. Through this, AI has become the breeding norm on the majority of dairy farms in Slovenia (22). The best bulls are used to obtain semen for AI and kept in quarantine conditions. Calves from herds with bull's mothers are selected by the Slovene cattle breeding selection service. These calves are kept in BCYB under quarantine conditions until the age of 12 months and then transferred to IC. Two ICs and two BCYBs have been established in Slovenia. The development of virological diagnostics in 1963 also enabled the routine diagnosis of BoHV-1 virus infections (18). The knowledge that BoHV-1 is also very successfully transmitted by infected semen was first introduced to control the disease in bulls in IC in the 1970s. In epizootiological terms, the prevention of BoHV-1 infection of bulls in ICs is very important. ICs were supplied with new bulls by young bulls that were bred in BCYBs or were brought from abroad. Before 1985, when VNT on microplates was introduced, VNT techniques for detecting antibodies did not allow testing of a large number of samples, and surveillance was limited to bulls in ICs and BCYBs.

Due to the increased milk needs in Yugoslavia in the 1960s, the intention was to increase milk production by importing high-milk breeds such as Holstein Friesian cows; they were imported from various European countries and from Israel. This breed was bred on state dairy farms, while traditional breeds such as Brown cattle and Simmental cattle were present in private farms. At that time, imported cattle were not tested for BoHV-1 infection, so virus could be introduced into the country with the import of BoHV-1 positive Holstein Friesian cattle. Herds with Holstein Friesian cattle were later detected heavily infected with the BoHV-1 in comparison to the lower detected prevalence in the herds of the private sector.

Between 1976 and 1984, 15.5% serologically positive samples were identified from state-owned farms, while from private herds, only 3.6% of animals reacted positively. By 1984, all tested state-owned herds, with from 500 to 1,500 dairy cows had been infected the BoHV-1 positive. The reason for this can be result of intensive traffic of breeding livestock between individual state farms without testing for BoHV-1 infection before movements. These results (Table 1) show that the infestation of Holstein Friesian cattle in state herds was significantly higher than in private sector herds, predominantly with Brown and Simmental breeds. Serological monitoring of cattle confirmed that BoHV-1 infection was present in all state-owned dairy farms and uncontrolled purchases of cattle from these farms were the main cause of infection for private herds.

Epidemiological analyses performed between 1985 and 1990 confirmed that BoHV-1 was widespread among Holstein Friesian cattle and less so among Simmental and Brown Swiss. After separation from Yugoslavia and the introduction of the democratic political system in Slovenia in 1991, most state farms with Holstein Friesian breed collapsed and were depopulated or privatised. Many cattle were culling or sold to private herds, uncontrollably spreading BoHV-1 to these farms (21).

After 1991 the number of imported cattle increased sharply, especially fattening. In period from 1991 to 1995, a total 46.237 fattening cattle were imported from the Czech Republic, Hungary, Germany and Poland and tested for BoHV-1 in quarantine. The percentage of detected BoHV-1 seropositive animals from these imported countries was decreasing (Table 3). This can be attributed to the fact that these countries have already started with implementation BoHV-1 eradication programs during this period, which was also reflected in exported calves intended for further fattening. Imported cattle for fattening was for slaughter, trading of these animals to other breeders was not officially allowed. From the results of serological tests in the bulls' mother herds in 1995 (Table 3), 25.6% of samples were positive for BoHV-1 antibodies. The percentage of positives animals was higher because herds of Holstein Friesian cattle, mostly purchased from former large BoHV-1 positive state-owned herds, were also included in the investigations. Because of this and the goal to protect the bulls in ICs and BCYBs, an agreement was reached together with the Veterinary administration and Livestock Selection Service of Slovenia in 1995 that only calves from BoHV-1 negative herds may enter BCYBs. Surveillance started in 1996 with annual serological control in herds of bull's mothers. A BoHV-1 negative herd status was granted, after serological examination of all breeding animals, confirmed all animals are negative to BoHV-1 antibodies.

Annual laboratory control of herds with bull's mothers was carried out until 2003. During this period, 78.751 cattle were tested with an annual incidence of positive animals from 0.03 to 1.4%. A herd in which BoHV-1 positive cattle had been detected was no allowed to send calves to BCYB and lost status of bull's mother herd. In 25 herds only one positive animal was confirmed, which was unusual according to the literature, which shows that when the BoHV-1 virus is actively circulating within an uninfected herd, most animals seroconvert in a short time (23).

The results of epidemiological inquiries from BTM samples in herds with only individual BoHV-1 serologically positive animals showed that they were mostly highly productive Holstein Friesian cattle purchased from state herds after their closing. In the infected herd, we confirmed a higher percentage of positive animals among the older animals than the young, which is also evidenced by the literature (23). The infection spreads particularly rapidly in the case of natural mating with a BoHV-1 positive bull (24, 25).

An important component of the dynamics of BoHV-1 in an individual herd is the reactivation of the virus, which is often due to stress and triggers further primary infections in the herd (26).

An interesting case was noted in a herd that consisted predominantly of Brown cattle. The only positive cow in this herd was Holstein Friesian, which was purchased 7 years previously in one of the state farms and had never infected any animal in contact. The cow was never vaccinated. Outbreaks occur due to reactivation of the virus or new introduction of the virus from outside of the herd (26). Several studies report extended periods with no evidence of BoHV-1 circulation in endemically infected herds (27).

In 2000 the veterinary service in Slovenia sought to draught legislation for a mandatory eradication program of BoHV-1 throughout the country, following the example of Austria, Germany and other European countries. Monitoring of BoHV-1 was carried out in BTM samples from 13,349 tested herds producing milk for public consumption. BoHV-1 antibodies were found in samples from 449 (3.4%) examined herds. A total of 22,330 cattle were in positive herds. The reason for the highest percentage of serologically positive animals in the Gorenjska region (6.2% of positive herds), with an average 15.2 herd size can be attributed to the method of breeding in this area. In this region communal mountain grazing system is practised during summer time, where the possibility of infection spread between herds is greatly increased, especially when natural mating is practised. While the Prekmurje region (0.9% of positive herds), with an average size of 19.7 cows covers the flat part of the country, with more closed type of breeding without tradition of communal grazing, to which the lowest percentage of infection can be attributed.

To obtain accurate data on the prevalence of BoHV-1 required for the preparation of the national programme for the eradication of BoHV-1, in 2006, an intensive epidemiological analysis of BoHV-1 was performed in Slovenia. For the first time, individual blood samples were taken from cattle over 24 months of age. A total of 79.9% of the cattle population from 35,991 farms was tested. BoHV-1 antibody-positive animals were found on 1,287 farms, which represents 3.6% of farms. Vaccinations against BoHV-1 were not performed in these herds. The highest percentage of infected farms (8.8%) was detected on region of Gorenjska where communal mountain grazing of young stock, grazing and mowing system of cattle breeding is practised.

In 2015, the Veterinary Administration adopted rules on the conditions for the recognition, acquisition and maintenance of the status of herds free from infectious bovine rhinotracheitis/infectious pustular vulvovaginitis (Uradni list RS, No. 55/15). This regulation specifies the conditions for obtaining BoHV-1 free status and for maintaining the status in accordance with Commission Decision 2004/558/EC and Council Directive 64/432/EEC. Slovenian legislation currently allows breeders to obtain the status of breeding free of BoHV-1 virus infection, but on a voluntary basis. All costs of sampling and laboratory tests are paid by the owners, without any financial compensation. Breeders are not sufficiently aware and do not believe in the benefits of raising cattle without BoHV-1 virus infection. The response from breeders is very poor. Currently, only one breeder with the official status of being free of BoHV-1 is recorded in the database of the Veterinary Administration.

BoHV1 control in Germany is based on two different strategies, which mainly depend on the initial BoHV1 sero-prevalences. In herds, regions, or federal states with low rates of BoHV1-infected animals, the so-called “conventional eradication” concept focuses on the selection of BoHV1-seronegative animals without vaccination. In regions with high BoHV1-sero-prevalences, eradication is based on immunisation with glycoprotein E (gE)-deleted marker vaccines and the subsequent selection of marker-negative animals. At the end of 2010, nationwide, 90.4% of the dairy and breeding herds in Germany were BoHV1-free (with or without vaccination), and in 6.3% of the herds, eradication was still in progress (28).

Although the presence of BoHV-1 virus infection in Slovenia has been serologically determined for many years, there are no frequent reports of clinical outbreaks of the disease on individual farms. In Europe, various eradication methods have been used, such as killing seropositive animals, lifelong vaccination of seropositive animals only or vaccination of all cattle in IBR/IPV positive breeding (29).

The number of samples sent for BoHV-1 infection investigations has declined sharply after 2007, with testing from 1,000 to 2,000 samples per year in Slovenia. This small number of not randomly selected animals each years was showed low detected prevalence, with no improvement in last two decades.

The efforts made by the veterinary and cattle breeding service for eliminating the virus in the past have brought us to the point at which the main question is whether we shall maintain the prevalence of the disease in its current state or decide to eradicate the disease gradually. Only BoHV-1 free ICs are not sufficient to control BoHV-1. Bull's mother herds with uncontrolled status pose a high risk of BoHV-1 spread into BCYBs and ICs.

Over the years, a great deal of money has been invested in the surveillance and control of BoHV-1 in Slovenia. Given the known prices of sampling and the prices of laboratory tests, they can be estimated to over €6,500,000. An important contribution of all past efforts is that the bulls in ICs and BCYBs are remaining free of BoHV-1 infection. Attempts of the veterinary profession to take a mandatory approach to eradicate BoHV-1 have not been successful so far in Slovenia. We are promoting many EU countries success storeys to our stakeholders and hope that soon an obligatory nation-wide eradication programme will be adopted.

## Data Availability Statement

The raw data supporting the conclusions of this article will be made available by the authors, without undue reservation.

## Author Contributions

IT and PH: conceptualisation and writing-original draught preparation. IT, DČ, PH, JM, and JS: samples collection organisation, writing-review, and editing. IT, DČ, and PH: methodology, investigation, and: formal analysis. All authors have read and agreed to the published version of the manuscript.

## Conflict of Interest

The authors declare that the research was conducted in the absence of any commercial or financial relationships that could be construed as a potential conflict of interest.
